# Testing a Deep Learning Algorithm for Detection of Diabetic Retinopathy in a Spanish Diabetic Population and with MESSIDOR Database

**DOI:** 10.3390/diagnostics11081385

**Published:** 2021-07-31

**Authors:** Marc Baget-Bernaldiz, Romero-Aroca Pedro, Esther Santos-Blanco, Raul Navarro-Gil, Aida Valls, Antonio Moreno, Hatem A. Rashwan, Domenec Puig

**Affiliations:** 1 Ophthalmology Service, Hospital Universitat Sant Joan, Institut de Investigació Sanitària Pere Virgili [IISPV], Universitat Rovira & Virgili, 43204 Reus, Spain; mbaget@gmail.com (M.B.-B.); mariaesthersantosblanco@gmail.com (E.S.-B.); raul_navarro_gil@hotmail.com (R.N.-G.); 2 Department of Computer Engineering and Mathematics, Universitat Rovira & Virgili, 43204 Reus, Spain; aida.valls@urv.cat (A.V.); antonio.moreno@urv.cat (A.M.); hatem.rashwan@gmail.com (H.A.R.); domenec.puig@urv.cat (D.P.)

**Keywords:** diabetic retinopathy, deep learning algorithm, convolutional neural networks, diabetic retinopathy screening

## Abstract

Background: The aim of the present study was to test our deep learning algorithm (DLA) by reading the retinographies. Methods: We tested our DLA built on convolutional neural networks in 14,186 retinographies from our population and 1200 images extracted from MESSIDOR. The retinal images were graded both by the DLA and independently by four retina specialists. Results of the DLA were compared according to accuracy (ACC), sensitivity (S), specificity (SP), positive predictive value (PPV), negative predictive value (NPV), and area under the receiver operating characteristic curve (AUC), distinguishing between identification of any type of DR (any DR) and referable DR (RDR). Results: The results of testing the DLA for identifying any DR in our population were: ACC = 99.75, S = 97.92, SP = 99.91, PPV = 98.92, NPV = 99.82, and AUC = 0.983. When detecting RDR, the results were: ACC = 99.66, S = 96.7, SP = 99.92, PPV = 99.07, NPV = 99.71, and AUC = 0.988. The results of testing the DLA for identifying any DR with MESSIDOR were: ACC = 94.79, S = 97.32, SP = 94.57, PPV = 60.93, NPV = 99.75, and AUC = 0.959. When detecting RDR, the results were: ACC = 98.78, S = 94.64, SP = 99.14, PPV = 90.54, NPV = 99.53, and AUC = 0.968. Conclusions: Our DLA performed well, both in detecting any DR and in classifying those eyes with RDR in a sample of retinographies of type 2 DM patients in our population and the MESSIDOR database.

## 1. Introduction

It is estimated that 463 million people worldwide suffer from diabetes mellitus (DM), a number that is predicted to rise to around 640 million by 2040 [1,2]. DM is one of the main causes of morbidity and mortality in the developed world, the organs mostly affected being the brain, the heart, the kidneys, and the eyes. Approximately 30% of diabetes patients end up developing some type of diabetic retinopathy (DR) [3,4], which is currently the leading cause of preventable vision loss and blindness in the working-age population [5].

The onset and progression of DR is related primarily to the duration of DM, along with other risk factors such as poor glycemic control, the type of treatments, and/or the presence of arterial hypertension. It is well known that early diagnosis of DR leads to a better response to treatment and a more positive prognosis [6].

All diabetes patients have the potential to develop DR, and, therefore, regular screening and early diagnosis are essential. It can be detected by fundus examination or by retinography, which is more commonly recommended and forms the basis of current DR screening protocols. All diabetes and ophthalmology associations currently recommend annual retinography screening for all diabetes patients from first diagnosis [7,8,9]. Screening programmes are most commonly carried out at ophthalmology units, where a patient’s retinas can be evaluated by an ophthalmologist.

Telemedicine systems are available across the developed world, where patients can attend their screening centre to have a photograph taken of their retinas and then the healthcare professionals can further examine them [10]. However, this is not considered to be cost-effective because the manual grading of each patient’s retinas requires a large number of specialists to be involved in it. In addition, current screening services are not able to keep up with the huge demand and, as a result, fewer than 50% of diabetes patients have regular examinations. In order to alleviate this problem, several automatic reading systems are being developed that use artificial intelligence-based algorithms that can detect DR. Currently available deep learning algorithms (DLA) have performed well in various DR classification tasks [11,12]. Most related works provide a binary classification distinguishing those patients who should be referred with a more than mild DR and those who do not have signs of DR. In addition, these DLA seem to be cost-effective when compared to manual grading, which is the current gold standard [13,14].

Using new deep learning techniques, we developed a DLA system that is able to classify retinal images into four different DR categories: no DR, mild DR, moderate DR, and severe DR [15,16]. The aim of the present study was to test a new DLA system for automatic reading of retinographies of Type 2 DM patients, with the ability to detect and classify a patient’s level of DR using the standard four categories of the International Clinical Diabetic Retinopathy severity scale (ICDR). The testing phase of our DLA was carried out by comparing its performance in detecting any type of DR (any DR) or referable DR (RDR) against the DR diagnoses and classification made by four masked retinal specialists. The sample of retinal images were taken from type 2 diabetes (Type 2 DM) patients extracted from records of our population, and the patients were different from those used to train and validate the DLA. In addition, we used images from the MESSIDOR database [17], which contains a collection of 1200 retinal images centred on the macula of diabetes patients. MESSIDOR is available on the web with the purpose of facilitating studies on computer-assisted diagnoses of DR.

We presented our preliminary results at ARVO 2020.

## 2. Subjects

### 2.1. Setting

The reference population of our Health Care Area (University Hospital Saint Joan, Tarragona, Spain) is 247,174. The total number of registered DM patients is 17,792 (7.1%). Our DR screening programme has been ongoing since 2007, when we started offering annual retinography to our Type 2 DM patients. The complete screening programme is described elsewhere [9].

### 2.2. Datasets

We used a sample size of 16,186 retinographies, of which 14,186 corresponded to 7164 patients from our screening programme during the period 1 January 2019 to 31 December 2019, and 1200 retinal images extracted from MESSIDOR. In addition, the EyePACS database was used to construct the multi-class DLA model for diabetic retinopathy [18].

### 2.3. Design

To validate and test the performance of this new DLA system, we compared the DR categories assigned by the DLA with those DR categories established by the four expert retina ophthalmologists.

### 2.4. Inclusion and Exclusion Criteria

Retinographies of Type 2 DM patients from our screening programme and MESSIDOR were included. Retinographies of other diabetes types, such as diabetes in pregnancy or T1DM patients, were excluded.

## 3. Material and Methods

### 3.1. Ethics and Consent

The study was carried out with the approval of the local ethics committee (approval no. 13-01-31/proj6) and in accordance with the revised guidelines of the Declaration of Helsinki. Patients gave signed consent after being informed about the objective of the study. Subjects were recruited according to the screening programme protocol, which uses a systematic technique.

### 3.2. Imaging Technique

One 45° field retinography was centred on the macula, taken by a non-mydriatic fundus camera (TOPCON^®^ TRC-NW6S). Retinal images were taken by the research assistants according to the regular procedure, which does not include dilation as routine. First, the reading was made by the DLA if the gradeability function considered the image to be of sufficient quality. Otherwise, immediate feedback was provided by the software and a new retinography was taken. A second reading was then made independently by four senior retina specialists masked from each other and from the DLA device output. Cohen’s Weighted Kappa (CWK) index was used to evaluate the degree of agreement, the results of which have been published previously [19]. The specialists met to discuss cases which they had not agreed on initially so they could achieve a consensus.

DR was diagnosed when microaneurysms, the first visible sign of DR, were present in the fundus photograph in the absence of other known causes of the changes [20,21]. DR was classified into 4 stages according to the International Clinical Diabetic Retinopathy severity scale (ICDR) [22]:

Level 0 = no DR.

Level 1= mild DR (only microaneurysms).

Level 2 = moderate DR (more than microaneurysms but less than severe).

Level 3= severe (more than 20 haemorrhages per quadrant or two quadrants with venous beading or one quadrant with intraretinal microvascular abnormalities) or proliferative DR (presence of new vessels elsewhere) or referable diabetic macular edema.

### 3.3. Model Construction

In our previously published work, we presented a set of recommended guidelines to construct a DLA for diabetic retinopathy classification [14]. Following this guide, our DLA created eye fundus images with a size of 3 × 640 × 640. The model consists of a convolutional neural network with 7 blocks of 2 layers each that progressively reduce the size of the data until it has a receptive field of 64 × 5 × 5 for feature extraction. Each layer is a stack of a 3 × 3 convolution with stride 1 × 1 and padding 1 × 1, followed by batch normalisation and a ReLU activation function. The final vector has a size of 64 values, which is obtained from a 4 × 4 average pooling stage. In the last layer, a linear classifier and a soft-max function use these 64 features to calculate the probability of each of the DR levels according to the ICDR scale. For optimisation of the parameters of this convolutional neuronal network, the quadratic weighted kappa is used as a loss function, because it is more appropriate for ordinal classification [13]. Details of the architecture can be found in this work [14]. The algorithm building comprised training, validation and testing phases.

### 3.4. Training, Validation and Testing the DLA

#### 3.4.1. Training

Two different retinal image datasets were used to train the model, with a total of 103,815 images: our own sample of 15,123 graded fundus images (different patients from the validation and testing phases) and 88,692 retinal images extracted from EyePACS [18]. There were 81,266 retinal images with no DR (level 0), 8771 with mild DR (level 1), 14,097 with moderate DR (level 2), and 4588 with severe or proliferative DR (level 3) in the training phase.

#### 3.4.2. Validation

Validation was carried out with 5000 retinographies from our population. The results of the DLA when detecting any DR were: sensitivity (S) = 0.967%, specificity (SP) = 0.976%, positive predictive value (PPV) = 0.836% and negative predictive value (NPV) = 0.996, and error type I = 0.024 and error type II = 0.004. The RDR results were: S = 0.998, SP = 0.968, PPV = 0.701, NPV = 0.928, error type I = 0.032, and error type II = 0.001.

#### 3.4.3. Testing

The test phase has now been carried out with a larger patient population to study the performance of our DLA on two different datasets. First, the DLA was given 14,186 retinal images from 7164 patients in our diabetic population to be classified according to the level of DR severity. Second, we tested the DLA with 1200 images extracted from the MESSIDOR dataset to repeat the operation.

The DLA includes a preliminary step named ‘gradeability’ that indicates the percentage of possibility of reading the retinal image. In cases of low gradeability, the DLA did not continue reading and the image was immediately discarded. These were cases of either poor image quality or not being sufficiently centred on the macula.

### 3.5. Statistical Methods

Data evaluation and analysis was carried out using SPSS 22.0 statistical software package at a significance of *p* < 0.05.

Characteristics of the patients with T2DM in this study were presented as means (standard deviation (SD)) or proportions.

The results of the DLA were compared to the results of the manual grading. To measure the effectiveness of the classification model, we used Cohen’s weighted Kappa (CWK) index. The results of the CWK were described with a 95% confidence interval. We calculated the overall agreement of the DLA in detecting any DR and RDR, which was published in a previous article [19].

The measures taken were sensitivity (S), specificity (SP), positive predictive value (PPV), and negative predictive value (NPV). Sensitivity was considered as the probability that the result of the DLA was positive when DR was present according to specialist grading, and specificity as the probability that the result of the DLA was negative when DR was not present according to specialist grading. Positive predictive value (PPV) was considered the probability that patients with a positive screening test based on the DLA truly had DR. Negative predictive value (NPV) was considered the probability that patients with a negative screening result based on the DLA truly did not have DR. To evaluate the discriminatory ability of our DLA to detect RDR, the area under the receiver operating characteristic curve (AUC) was calculated. Finally, we determined the accuracy or diagnostic effectiveness, which is expressed as a proportion of correctly classified subjects. Accuracy is affected by prevalence, irrespective of sensitivity or specificity. The diagnostic accuracy of a particular test increases as the disease prevalence decreases, which was not the case in the present study.

## 4. Results

### 4.1. Description of Sample Size

We had a sample size of 14,327 retinal pictures from our population, corresponding to 7164 patients randomly taken from our screening programme during the period 1 January 2019 to 30 December 2019. The main demographic and clinical data are given in Table 1.

### 4.2. Testing the DLA in Our Population Cohort

The gradeability test allowed the reading of 14,186 retinographies from our sample of 14,327 diabetes patients and eliminated 141 retinographies (0.98%) for poor image quality. This set of 14,186 retinal images was read and classified by the DLA model, obtaining the following results: most images (11,827 images; 83.4%) did not show DR, and only 2321 images showed any type of DR (16.6%). From that, we had 788 images with mild DR (5.5%), 984 images with moderate DR (6.9%), and 597 images with severe and proliferative DR (4.2%).

The performance of our DLA, in comparison with the categories given by the four retina specialists, is shown in Table 2. The algorithm correctly detected 11,816 (99.9%) of those retinographies without DR and only misclassified 11 into the mild DR category. It correctly classified 732 (94.3%) retinographies with mild DR, and 36 were misclassified as not having DR and 10 as having moderate DR. The algorithm correctly identified 940 (95.5%) retinographies with moderate DR and misclassified 9 (0.09%), 32 (3.2%), and 3 (0.03%) that were graded by the ophthalmologists as having no, mild, or severe DR, respectively. Lastly, the algorithm correctly classified 516 (86.4%) retinographies into the severe category and misclassified 4 (0.6%), 13 (2.1%), and 64 (10.8%), which were considered as having no, mild, or moderate DR, respectively.

In Table 3 we describe the classification according to no DR against any DR and no DR plus mild DR against referable DR. Thus, from 11,827 images with no DR, the DLA correctly classified 11,816 (99.9%) and showed 11 false positives (0.09%). All these pictures were misclassified in the mild DR category. This is not a significant problem as these patients would be referred to an ophthalmologist who could then rule out the presence of DR. When testing the ability to detect the presence of any type of DR, the DLA had 49 false negatives (0.2%), all of them being mild DR, showing fewer than five microaneurysms. In the mild stage of DR, visual acuity is not usually compromised, and we can assume that these patients would be picked up in other check-ups in case of progression of their DR.

The performance of the DLA in differentiating the photographs with DR from these without was: ACC = 99.75 (99.65–99.82), S = 97.92 (97.26–98.46), SP = 99.91 (99.83–99.95), PPV = 98.92 (98.07–99.40), NPV = 99.82 (99.76–99.86), error type I = 0.0009, error type II = 0.004, and AUC = 0.983.

The DLA correctly identified 1523 retinographies that presented referable diabetic retinopathy (RDR) (96.7%) and differentiated them from 12,595 which had non-referable DR. In 10 (0.08%) cases, the DLA identified RDR when it was not present and misclassified 58 (3.7%) cases as not having RDR when it was present.

The performance of the DLA in differentiating the photographs without RDR from those with RDR was: ACC = 99.66 (99.55–99.75), S = 96.7 (95.69–99.49), SP = 99.92 (99.85–99.96), PPV = 99.07 (98.28–97.46), NPV = 99.71 (99.63–99.78), error type I = 0.0041, error type II = 0.033, and AUC = 0.988.

### 4.3. Testing the DLA with MESSIDOR Database

The gradeability test performed by the DLA on the 1200 retinographies from MESSIDOR found all of them gradable (100%). This was due to the high quality of all the retinal images in that dataset. After applying the DLA classification on the 1200 pictures, 625 images (52%) did not show DR (level 0), and 575 images showed some type of DR. There were 197 images with mild DR or level 1 (16.4%), 130 images with moderate DR or level 2 (10.8%) and 248 images with severe or proliferative DR or level 3 (20.6%).

Table 4 displays the performance of the algorithm when classifying all the retinographies from the MESSIDOR database into their different types of diabetic retinopathy DR. The algorithm correctly classified 94.5%, 87.7%, 81.1%, and 95.6% of those retinographies with no, mild, moderate, and severe DR, respectively. In those retinographies with severe DR, the algorithm only misclassified 11 (4.4%) cases as having a lower degree of DR: four in the mild category and seven in the moderate category.

Table 5 shows the performance of the DLA in differentiating those images with an absence of DR from those with some type of DR. In addition, it shows the performance of the DLA in differentiating those images with mild or no DR (non-referable) from those with moderate or severe DR (referable).

In Table 5, we can see the results achieved by the DLA classifier when analysing the retinographies of the MESSIDOR database. In case (A), the algorithm identified 545 retinographies that presented some type of diabetic retinopathy (97.4%) and differentiated them from 610 (94.6%) that were normal. In 35 cases, the DLA identified DR when it was not present (false positives: 5.4%), and in 15 cases identified no DR when it was present (false negatives: 2.6%). The performance of our algorithm in differentiating the photographs with some type of DR from those without DR was: ACC = 94.79 (93.38–95.98), S = 97.32 (95.62–98.49), SP = 94.57 (92.53–96.19), PPV = 60.93 (53.04–68.28), NPV = 99.75 (99.60–99.85), error type I = 0.054, error type II = 0.026, and AUC = 0.959.

In case (B), the algorithm identified 371 retinographies that presented referable diabetic retinopathy (94.7%) and differentiated them from 801 (99.1%) which had non-referable diabetic retinopathy. In seven cases, the DLA identified referable diabetic retinopathy when it was not present (false positives: 0.86%), and in 21 cases it identified non-referable diabetic retinopathy when it was present (false negatives: 5.3%). The performance of our DLA in differentiating RDR photographs from those without was: ACC = 98.78 (97.99–99.32), S = 94.64 (91.93–96.65), SP = 99.14(98.24–99.65), PPV = 90.54 (82.06–95.24), NPV = 99.53 (99.29–99.69), error type I = 0.009, error type II = 0.053, and AUC = 0.968.

## 5. Discussion

The aim of the present study was to test a new DLA system for automatic reading of retinographies of Type 2 DM patients, with the ability to detect and classify a patient’s level of DR using the standard 4 categories of ICDR scale. The mathematical formulation and specific details of the design of this DLA algorithm were published in a specialised journal [14]. The DLA system was especially designed to ease the burden of screening for DR in the ophthalmology unit.

The DLA classifier was built in three phases: training, validation, and testing [14]. In each phase, we used retinographies of different Type 2 DM patients. During the training phase, up to 103,815 pictures were taken from both our reference population and the EyePACS database. The reason for using such a large number of retinographies extracted from different populations was to better adjust the algorithm and minimise the over-fitting [23].

The validation phase conducted during the construction of the DLA was made using 5000 retinographies from Type 2 DM patients in our population. The sensitivity (S) and specificity (SP) of the DLA in correctly identifying any DR were 0.96% and 0.97%, respectively, while the error type I was 0.024 and the error type II was 0.004. Likewise, the RDR results were: S = 0.99%, SP = 0.96%, error type I = 0.032, and error type II = 0.001. Most of the small number of false negatives that we obtained had mild DR (86.3%) and only 3.4% showed severe DR.

In this paper we have conducted an extensive test of this DLA classifier. We have evaluated the efficacy of the algorithm by differentiating normal from pathological retinographies and, subsequently, by classifying those eyes with no or mild DR from those with RDR in two different samples, our reference population, and the MESSIDOR database. However, before the retinographies were analysed, they underwent a gradability test that is incorporated into our DLA to improve readability. It has an initial filter that only allows the retinographies to be further analysed if they are well centred on the macula and of sufficient quality to be read. The gradeability test allowed the algorithm to read 99% and 100% retinographies from the two sample origins, respectively. Regarding the population sample, the gradeability test initially discarded 141 retinographies (0.9% of the total) that were then not analysed, giving the algorithm greater reliability. 

The first sample used to test the DLA included 14,186 retinographies from 7164 randomly selected Type 2 DM patients in our population and the second sample consisted of 1200 retinographies extracted from MESSIDOR, which was created using images from three different hospitals in France that had used different cameras and settings. The images were classified following the International Classification of Diabetic Retinopathy guidelines. We chose the MESSIDOR dataset due to the high quality of its photographs and its use of standard classifications.

The DLA correctly identified 99.9% (our population) and 94.6% (MESSIDOR) of the retinographies without DR. False positives yielded from the two samples were 0.09% and 5.4%, respectively. This is not a significant problem, since these patients would be referred to an ophthalmologist who would then rule out the presence of DR.

When testing the ability to detect the presence of any DR, DLA was successful in 99.5% and 97.4% of the two samples, respectively. False negatives yielded were 2% and 2.6%, respectively, of which all presented with fewer than five microaneurysms. In the mild stage of DR, visual acuity is not usually compromised, and we can assume that these patients would be identified in future check-ups if their DR progressed.

Regarding the ability of the DLA to classify those retinographies with RDR, it identified 96.7% and 94.7%, respectively. The false positives yielded were 0.08% and 0.86% and the false negatives were 3.7% and 5.3%, respectively. Most of the retinographies that the DLA failed to identify as having RDR had moderate DR (81% and 95%, respectively).

Regarding the ability of the DLA to identify retinographies with any DR and RDR, we found a higher proportion of false positives and false negatives when testing the retinographies from MESSIDOR compared to our own population. This might be due to differences in pixel definition of the pictures, as the dataset was compiled using different cameras, rather than our own images that were taken with the same model of non-mydriatic fundus camera. However, it is fair to conclude that the performance of our DLA in differentiating normal retinographies from those with any DR was very high.

In recent years, several research groups have developed some DLAs capable of reading retinal images of DM patients by identifying those with RDR. The first to obtain marketing approval by the FDA was IDx-DR^®^ (Coralville, IA, USA), which analyses retinographies taken with a non-mydriatic Topcon NW400 retinal camera and identifies images of concern. The RDR in a prospective study of 900 patients in a primary care setting were 91% (S) and 84% (SP). Its gradeability rate was 96.1% [24]. Another DLA that has obtained the CE Class IIa mark and FDA approval is EyeArt^®^ (Eyenuk, Woodland Hills, CA, USA) [25], which uses a Canon CR-2 plus AF and gave a gradeability rate of 87%, increasing to 97% if the pupil had been dilated. EyeArt was evaluated using a large number of retinal images from more than 100,000 consecutive DM patients and was subsequently independently validated by the National Health System (NHS) in the UK. When identifying RDR, results showed 91.3% (S) and 91.1% (SP) with an AUC of 0.965 [16,26]. Retmarker^®^ (retmarker SA, Taveiro, Portugal) has also obtained CE Class IIa mark approval. It was evaluated using more than 100,000 retinographies from 20,258 consecutive patients from the NHS in the UK. It gave 73% (S) and 85% (SP) when detecting any DR and RDR, respectively [16]. Lastly, RetCAD, an algorithm trained to detect DR and age macular degeneration, achieved an S of 90.1% and an SP of 90.6% with respect to RDR disease. It was validated using the MESSIDOR database [27].

It is fair, then, to again conclude that our DLA performed very well in both samples in detecting RDR, compared to those that have already been approved by the FDA or that have obtained the CE Class IIa mark. Identifying those patients with a moderate or higher level of DR is very important to be able to refer them to the ophthalmologist for treatment. Our DLA is not only capable of identifying the referable cases but also to distinguish between the moderate and severe levels as it classifies the images in the 5-level ICDR scale [19].

All currently available DLAs make errors in detection and classification; therefore, we think it is important to find a consensus with respect to the classification of DR using deep learning criteria, besides the clinical criteria that are already established. Takahashi et al. [28] proposed a novel way of grading DR, based on modifying Davis [29], which has three stages: (1) simple diabetic retinopathy, (2) pre-proliferative diabetic retinopathy, and (3) proliferative diabetic retinopathy. Furthermore, we think that an independent validation of the screening algorithms would be advisable before their clinical application.

The strengths of the present study are that it has used a real population to test a DLA system with no prior selection of patients. In addition, it has been tested with the MESSIDOR database, which provides retinographies that are correctly graded and of good image quality.

A limitation of our study is that we tested our DLA using retinographies taken by only one type of non-mydriatic fundus camera, so further study is essential into whether other models might change the results. In addition, our algorithm should be tested on larger samples of patients and with greater ethnic variety. Lastly, it is worth mentioning the capability of this DLA of performing an initial analysis of the image to determine if it is gradable or not (i.e., if the image is of a high enough quality or must be repeated. Using this procedure, the DLA eliminated 141 retinographies (0.9% of the total) due to poor image quality.

## 6. Conclusions

Our DLA can be used as a reliable diagnostic tool to ease the screening for DR, especially when it might be difficult for ophthalmologists or other professionals to identify it. It can identify patients with any DR and those that should be referred. Reducing the time taken to read images, and, at the same time, the cost of screening, will increase the number of patients who can be screened annually for DR.

## Figures and Tables

**Table 1:** Baseline characteristics from both our reference population and MESSIDOR database that were used to test our deep learning algorithm built to detect any type of diabetic retinopathy (DR) and referable DR.

Characteristics	Our Population	MESSIDOR
Patient demographics		
No. of unique individuals	7164	874
Age, mean (SD) (years)	67.3 (12.01)	57.6 (15.9)
Gender (male %)	54.6	57.4
Retinal images		
No. of images	14,186	1200
No. of ophthalmologists	4	4
No. of grades per image	4	4
Gradeability rate (%)	99.00%	100.00%
DR distribution classified by the ophthalmologists (reference standard). No, (%)		
No DR (level 0)	11,827 (83.4)	625 (52.0)
Mild DR (level 1)	778 (5.5)	197 (16.4)
Moderate DR (level 2)	984 (6.9)	130 (10.8)
Severe or proliferative DR (level 3)	597 (4.2)	248 (20.6)
Referable DR	1503 (10.6)	378 (31.5)

**Table 2:** Performance of our DLA in detecting and classifying each type of DR with respect to the classification carried out by the four senior retina specialists.

		Predicted by the DLA			
		No DR	Mild DR	Moderate DR	Severe DR
Diagnosed by Ophthalmologists	No DR	11,816	11	0	0
	Mild DR	36	732	10	0
	Moderate DR	9	32	940	3
	Severe DR	4	13	64	516
Total		11,865	788	1014	519

**Table 3:** (A) Results given by the DLA according to its ability to differentiate images with an absence of DR from those with any type of DR. (B) Results of the DLA in differentiating images with no or mild DR (non-referable DR) from those with moderate or severe DR (referable DR).

**A**		**Predicted by the DLA**	
		**No DR **	**Any DR**
Diagnosed by ophthalmologists	No DR	11,816	11
	Any DR	49	2310
		11,865	2321
**B**		**Predicted by the DLA**	
		**No DR + Mild DR**	**Referable DR**
Diagnosed by ophthalmologists	No DR + mild DR	12,595	10
	Referable DR	58	1523
		12,653	1533

**Table 4:** Performance of our DLA in detecting and classifying each type of DR in 1200 pictures with respect to their classification by four senior retina specialists.

		Predicted by the DLA			
		No DR	Mild DR	Moderate DR	Severe DR
Predicted by MESSIDOR	No DR	610	35	0	0
	Mild DR	13	143	7	0
	Moderate DR	2	15	116	10
	Severe DR	0	4	7	238
Total		625	197	130	248

**Table 5:** Classification of results achieved by the algorithm when analysing the retinographies extracted from the MESSIDOR database. (A) Any DR case (B) referable DR case.

**A**		**Predicted by the DLA**	
		**No DR**	**Any DR**
Predicted by MESSIDOR	No DR	610	35
	Any DR	15	545
		625	575
**B**		**Predicted by the DLA**	
		**No DR + Mild DR**	**Referable DR**
Predicted by MESSIDOR	No DR + Mild DR	801	7
	Referable DR	21	371
		822	378

## Data Availability

The database used and analysed is available from the corresponding author on research request.
